# Association Between Statin Use and Prevalence of Exercise-Related Injuries: A Cross-Sectional Survey of Amateur Runners in the Netherlands

**DOI:** 10.1007/s40279-017-0681-7

**Published:** 2017-01-30

**Authors:** Esmée A. Bakker, Silvie Timmers, Maria T. E. Hopman, Paul D. Thompson, André L. M. Verbeek, Thijs M. H. Eijsvogels

**Affiliations:** 1 Department of Physiology (392), Radboud Institute for Health Sciences, Radboud University Medical Center, P.O. Box 9101, 6500 HB Nijmegen, The Netherlands; 2 Department of Health Evidence, Radboud Institute for Health Sciences, Radboud University Medical Center, Nijmegen, The Netherlands; 3 Division of Cardiology, Hartford Hospital, Hartford, CT USA; 4 Research Institute for Sport and Exercise Sciences, Liverpool John Moores University, Liverpool, UK

## Abstract

**Background:**

HMG-CoA reductase inhibitors (statins) are the first-choice therapy for primary prevention of cardiovascular disease. Some maintain that statins cause adverse musculoskeletal outcomes in highly active individuals, but few studies have examined the effects of statins on exercise-related injuries.

**Objective:**

We sought to compare the prevalence of exercise-related injuries between runners who do or do not use statins.

**Methods:**

Amateur runners (*n* = 4460) completed an extensive online questionnaire on their exercise patterns and health status. Participants replied to questions on the prevalence of exercise-related injuries in the previous year. Injuries were divided into general injuries, tendon- and ligament-related injuries, and muscle-related injuries. Participants were also queried about statin use: the type of statin, statin dose, and duration of treatment. Runners were divided into statin users, non-statin users with hypercholesterolemia, and controls for analysis.

**Results:**

The crude odds ratios (ORs) for injuries, tendon- or ligament-related injuries, and muscle-related injuries in statin users compared with controls were 1.14 (95% confidence interval [CI] 0.79–1.66), 1.10 (95% CI 0.71–1.72), and 1.15 (95% CI 0.69–1.91), respectively. After adjustment for age, sex, body mass index (BMI), and metabolic equivalent of task (MET) h/week of exercise, the ORs were 1.11 (95% CI 0.76–1.62), 1.06 (95% CI 0.68–1.66), and 0.98 (95% CI 0.58–1.64), respectively. Similar effect measures were found when comparing non-statin users with hypercholesterolemia and controls.

**Conclusion:**

We did not find an association between statin use and the prevalence of exercise-related injuries or tendon-, ligament-, and muscle-related injuries. Runners receiving statins should continue normal physical activity without concern for increased risk of injuries.

## Key Points

**Table:** 

There is no evidence of an association between statin use and the prevalence of exercise-related injuries in amateur runners.
Runners receiving statins should continue normal physical activity without concern for increased risk of injuries as statin use and exercise training both improve their cardiovascular risk profile.

## Introduction

HMG-CoA reductase inhibitors (statins) are the first-choice therapy for the primary prevention of cardiovascular disease (CVD) since they reduce all-cause and cardiovascular mortality and the risk of non-fatal cardiovascular events [1, 2]. Clinical trials examining the efficacy of statins seldom report statin-associated adverse events, and a meta-analysis did not find an increased risk of myopathy, fatigue, muscle aches, or rhabdomyolysis or an increase in creatine kinase concentrations >10 × the upper limit of normal in the statin arm compared with the placebo arm [3]. However, statin-associated side effects might be incompletely reported, and randomized trials may lack the right study design or be underpowered to detect musculoskeletal side effects [4].

A meta-analysis found a statistically significant decrease in absolute risk difference in myopathic symptoms (risk difference –0.4%; 95% confidence interval [CI] –0.5 to –0.3) and muscle aches (risk difference –0.6% [95% CI –1.1 to –0.1]) with low- versus high-intensity statin therapy. However, only 57% of the myopathic symptoms and 16% of the muscle aches were dose related [3]. A large observational study including 46,249 individuals showed a 19% increased risk of musculoskeletal disorders (odds ratio [OR] 1.19; 95% CI 1.08–1.30) in statin users compared with age-, sex-, and comorbidity-matched controls. Specifically, arthropathies, musculoskeletal injuries, and pain are more commonly observed in statin users than in matched controls [5]. An additional observational study revealed that statin use in individuals without arthritis is associated with a significantly higher prevalence of musculoskeletal pain in any region, the lower back, and the lower extremities (prevalence ratios 1.33 [95% CI 1.06–1.67]; 1.47 [95% CI 1.02–2.13]; 1.59 [95% CI 1.12–2.22], respectively) [6]. Additionally, case reports suggest that statins may cause tendon injuries, including tendinitis and tendon rupture [7, 8]. These observations suggest that muscular and tendinous injuries may be more prevalent in statin users. There is also evidence that statin-related side effects are more prevalent in physically active individuals. For example, the PRIMO (PRedIction of Muscular Risk in Observational Conditions) study reported myalgia in 10.5% of subjects overall, but the rate was 14.7% in patients practicing an “intense form of sport” [9]. Statin intolerance has also been reported in 22 professional athletes with low-density lipoprotein receptor defects, only six of whom could tolerate statins despite multiple attempts with the five statins available at that time [10]. Creatine kinase levels 24 h after a 42-km footrace were 38% higher in amateur runners receiving statins than in non-statin-treated controls [11]. Whether physically active individuals receiving statins are more likely to develop exercise-related injuries is unknown.

The aim of this study was to compare the prevalence of exercise-related injuries between statin-using runners and runners not using these medications. For this purpose, we studied participants in the 15-km ‘Seven Hills Run’ in Nijmegen, one of the largest road running races in the Netherlands. We hypothesized that statin-using runners would demonstrate an increased risk for muscle-, tendon-, and ligament-related sport injuries.

## Method

This cross-sectional analysis was performed as part of the Nijmegen Exercise Study (http://www.runningresearch.nl). This study is a large prospective study examining the impact of physical activity on health, quality of life, and the development and progression of chronic diseases. Amateur athletes participating in the Seven Hills Run were recruited by newsletter and internet advertisements. All Dutch-speaking adults participating in the 2014 Seven Hills Run were eligible to respond to the online questionnaire. Potential subjects provided written informed consent before they could access the online questionnaire, as approved by The Local Committee on Research Involving Human Subjects of the region Arnhem and Nijmegen, the Netherlands. Participants were asked about their general characteristics, lifestyle, medical history, and medication use.

### Questionnaire

The online questionnaire inquired in detail about general characteristics, lifestyle factors, medical history, and medication use. General characteristics included age, sex, weight, and height. Lifestyle factors were the average number of sleeping hours per night and the amount of habitual physical activity. The latter was estimated using the SQUASH questionnaire [12], which is divided into different domains (occupation, leisure time, household, and transportation) and asked participants about the duration and intensity of their weekly activities over past months. Weekly activities were converted to the average amount of metabolic equivalent of task (MET) hours per week spent on exercise. MET hours were calculated by multiplying the MET values of each sports activity by exercise duration (in h) [13]. The medical history contained questions about physician-made diagnoses of CVD, hypertension, hypercholesterolemia, and diabetes mellitus as well as the types and doses of medication used in the past year.

### Statin Use

Participants were asked whether they used atorvastatin (Lipitor or Atorab), fluvastatin (Lescol), pravastatin (Selektine), rosuvastatin (Crestor), simvastatin (Zocor), and the combination drugs ezetimibe/simvastatin (Inegy) and pravastatin/fenofibrate (Pravafenix) or any other cholesterol-lowering drugs. If participants used statins or combination drugs, they were classified as statin users. Participants with normal cholesterol values were classified as controls. Non-statin users with physician-diagnosed hypercholesterolemia were classified as non-statin users with hypercholesterolemia. Information on the intake frequency, statin dose, and duration of use was collected. To quantify a potential dose–response relationship, the dose of drug was standardized to ‘atorvastatin’ equivalence in which 1 equivalent = rosuvastatin 2.5 mg = atorvastatin 5 mg = simvastatin 10 mg = lovastatin 20 mg = pravastatin 20 mg = fluvastatin 40 mg [14, 15].

### Exercise-Related Injuries

Participants were questioned about their injuries in the previous year. If an injury had occurred, participants were asked to specify whether these injuries concerned muscle injuries (e.g., hamstring injury, back complaints, golfer’s elbow, calf strains), tendon or ligament injuries (e.g., Achilles tendon injuries, ankle sprain, groin injury, iliotibial band syndrome, knee ligaments injuries, plantar fasciitis, tennis elbow, or other tendon injuries), or other types of injuries (e.g., shin splint, hip injury, chondropathy, bursitis). Participants were also asked to report the duration of their injury and their need for medical help.

### Data Analysis

Differences in characteristics between statin users, non-statin users with hypercholesterolemia, and controls were assessed by descriptive statistics. Continuous variables are reported as mean ± standard deviation or median with 25th and 75th quartiles (Q_25_–Q_75_). Differences were tested with one-way analysis of variance (ANOVA) for normally distributed variables and by Kruskal–Wallis one-way ANOVA for variables that were not normally distributed. Categorical data were reported in proportions and tested with the chi-squared test.

To evaluate the relation between statin use and sport injuries, logistic regression was performed to determine ORs and adjust for possible confounders. Variables where classified as confounders if they satisfied the criteria for confounding [16]. Adjusted ORs were calculated with a 95% CI for the occurrence of sport injuries, tendon- or ligament-related injuries, and muscle-related injuries, for statin users and non-statin users with hypercholesterolemia compared with controls. All statistical analyses were performed using SPSS 20.0 (SPSS, Chicago, IL, USA) software. *P* values of <0.05 were considered statistically significant.

## Results

### Study Population

**Fig. 1:**
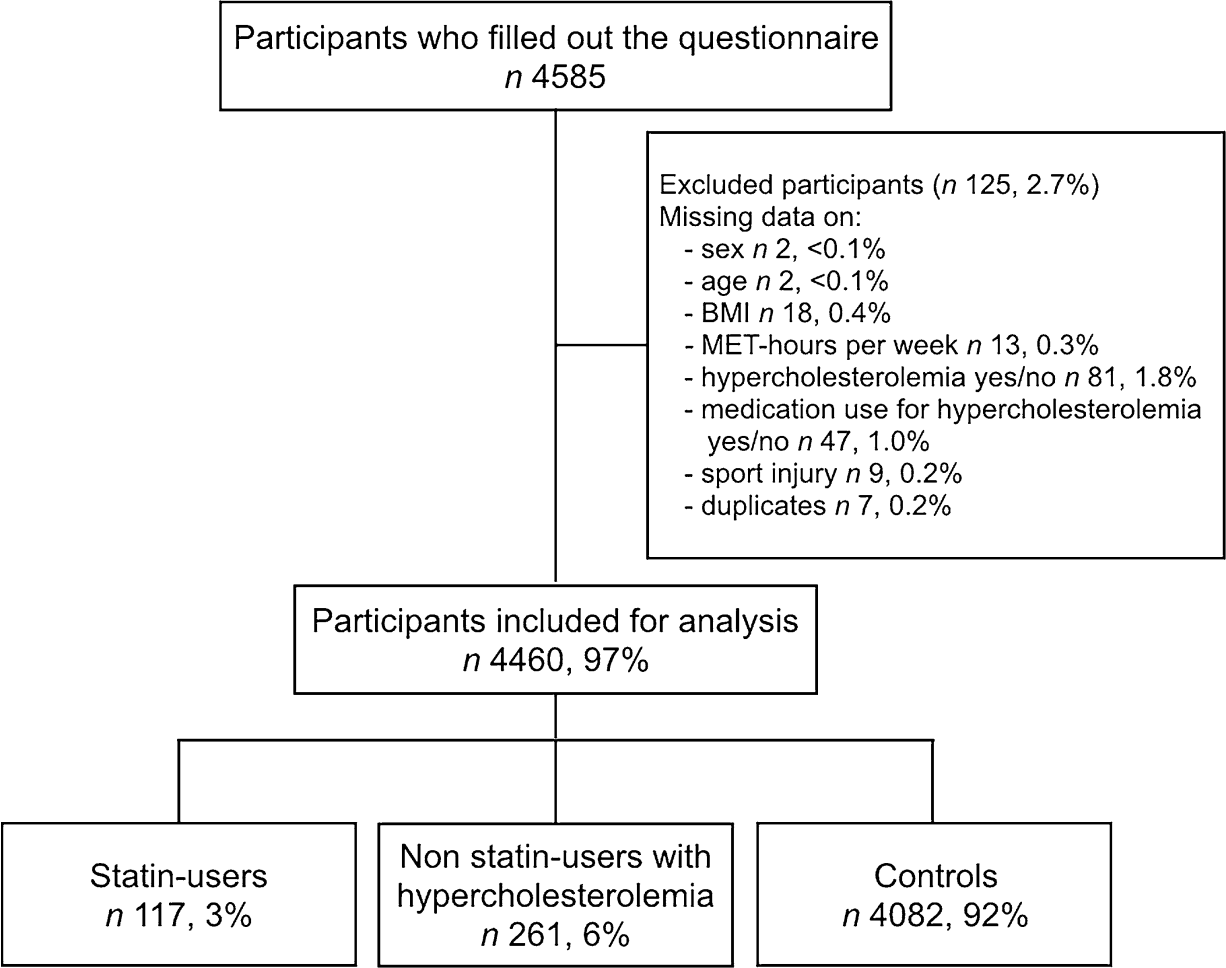
Flowchart of the enrolment of the study population. *BMI* body mass index, *MET* metabolic equivalent of task

In total, 4585 participants completed the questionnaire. After exclusion of participants with missing data, 4460 cases (97%) were available for analysis. In total, 378 runners reported a diagnosis of hypercholesterolemia; 117 of these respondents used statins (Fig. 1).

**Fig. 2:**
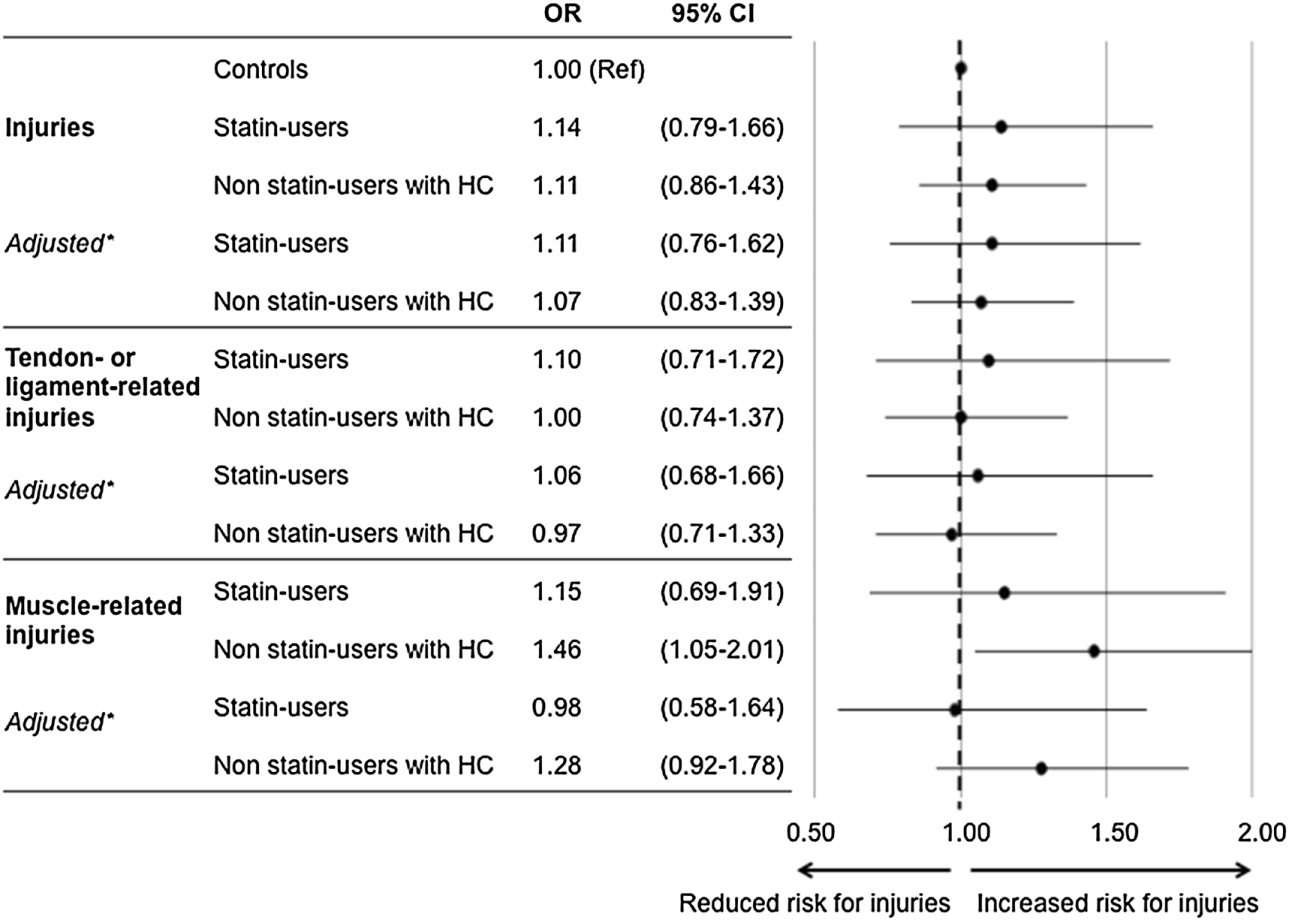
Crude and adjusted odds ratios of total injuries, tendon- or ligament-related injuries, and muscle-related injuries. *BMI* body mass index, *CI* confidence interval, *HC* hypercholesterolemia, *MET* metabolic equivalent of task, *OR* odds ratio. *Adjusted for age, sex, BMI, and MET hours per week

Compared with participants in the other two groups, statin users had a slightly higher body mass index (BMI), were a few years older, and were more likely to be men (all *p* < 0.001). CVD, hypertension, and diabetes mellitus were more prevalent in statin users than in the other two groups (*p* < 0.001). Lifestyle factors, such as running distance per training, and average number of sleeping hours per night, did not differ between the three groups (Table 1). MET hours spent per week on exercise was significantly higher in non-statin users with hypercholesterolemia compared to controls.

**Table 1:** Characterisation of 4460 participants divided into three groups relating to statin use and the presence of hypercholesterolemia

	Statin users (*N* = 117)	Non-statin users with hypercholesterolemia (*N* = 261)	Controls (*N* = 4082)	*p* value
Age (years)	56 ± 8	54 ± 9	46 ± 12	<0.001
Sex (male)	94 (80)	198 (76)	2337 (57)	<0.001
BMI (kg/m^2^)	24.6 ± 2.4	23.9 ± 3.5	23.2 ± 2.7	<0.001
Exercise (MET h/week)	32 (18–50)	34 (24–48)	32 (20–48)	0.042
Running distance per training session (km)^a^	10 (7–11)	10 (8–11)	10 (8–11)	0.465
Sleeping hours (per night)	7.0 ± 0.9	7.0 ± 0.9	7.0 ± 0.8	0.437
General health				
CVD^b^	35 (30)	26 (10)	128 (3)	<0.001
Hypertension	47 (40)	65 (25)	365 (9)	<0.001
DM	14 (12)	6 (2)	38 (1)	<0.001
Injuries				
Total injuries	48 (41)	105 (40)	1544 (38)	0.590
Tendon/ligaments	26 (22)	54 (21)	840 (21)	0.910
Muscle	18 (15)	49 (19)	559 (14)	0.066
Other	15 (13)	28 (11)	477 (12)	0.829
Injury duration (weeks)	6 (3–12)	5 (2–10)	5 (3–10)	0.56
Need for medical help	36 (31)	77 (30)	1076 (26)	0.570

Data are presented as mean ± SD, median (Q_25_–Q_75_), or *n* (%)

*BMI* body mass index, *CVD* cardiovascular disease, *DM* diabetes mellitus, *MET* metabolic equivalent of task

^a^1273 (29%) are missing values

^b^Myocardial infarction, heart failure, stroke, thrombosis, and atrial fibrillation

### Statin Use and Injury Prevalence

Simvastatin (57%) and atorvastatin (25%) were the most frequently used statins. The median potency of statin used by the participants was 4 (Q_25_ 2–Q_75_ 4). The duration of statin use was 0–2, 3–9, and >10 years in 30, 39, and 22% of the cases, respectively (Table 2).

**Table 2:** Quantification of the different types of statins, the effective dose, and the duration of statin use in participants using statins (*n* = 117)

Characteristics	Prevalence^a^
Statin type	
Atorvastatin (Lipitor or Atorab)	29 (25)
Ezetimibe/simvastatin (Inegy)	2 (2)
Fluvastatin (Lescol)	2 (2)
Pravastatin (Selektine)	4 (3)
Pravastatin/fenofibrate (Pravafenix)	1 (1)
Rosuvastatin (Crestor)	12 (10)
Simvastatin (Zocor)	67 (57)
Median expected potency of atorvastatin equivalents^b^	4 (Q_25_ 2–Q_75_ 4)
Duration of usage (years)	
0–2	34 (29)
3–9	45 (39)
≥10	27 (23)

*Q*_*25*_ 25th quartile, *Q*_*75*_ 75th quartile

^a^Data are presented as *n* (%) unless otherwise indicated

^b^One atorvastatin equivalent (5 mg) = rosuvastatin 2.5 mg = simvastatin 10 mg = lovastatin 20 mg = pravastatin 20 mg = fluvastatin 40 mg

Of the statin users, 41% reported an injury in the past year: tendon- or ligament-related sport injuries 22%, muscle-related injuries 15%, and other injuries 13%. The majority (30%) of the statin users reported one injury and 10% reported two injuries. Injury duration and the need for medical help did not differ between the three groups. Compared with statin users, injury prevalence was slightly but insignificantly lower in non-statin users with hypercholesterolemia (40%) and controls (38%) (Table 3).

**Table 3:** Quantification of 1697 sport injuries in the three study groups

	Statin users (*N* = 117)	Non-statin users with hypercholesterolemia (*N* = 261)	Controls (*N* = 4082)
Sport injuries	48 (41)	105 (40)	1544 (38)
Tendon or ligaments	26 (22)	54 (21)	840 (41)
Achilles tendon	13 (11)	23 (9)	331 (8)
Ankle sprain	1 (1)	7 (3)	125 (3)
Groin injury	4 (3)	2 (1)	65 (2)
Iliotibial band syndrome	5 (4)	3 (1)	128 (3)
Knee ligaments	2 (2)	7 (3)	81 (2)
Plantar fasciitis	2 (2)	8 (3)	83 (2)
Tendon injury	2 (2)	7 (3)	92 (2)
Tennis elbow	0 (0)	0 (0)	14 (0)
Other	1 (2)	7 (3)	107 (3)
Muscles	18 (15)	49 (19)	559 (14)
Golfer’s elbow	0 (0)	1 (0)	3 (0)
Hamstrings	7 (6)	14 (5)	193 (5)
Back complaints	6 (5)	11 (4)	185 (5)
Calf strains	4 (3)	9 (3)	59 (1)
Other	1 (1)	8 (3)	105 (3)
Other	15 (13)	28 (11)	477 (12)
Shin splint	1 (1)	7 (3)	82 (2)
Hip injury	4 (3)	5 (2)	127 (3)
Chondropathy	7 (6)	7 (3)	124 (3)
Bursitis	1 (1)	5 (2)	41 (1)
Other	3 (6)	10 (10)	160 (10)

Data are presented as *n* (%)

### Injury Risk Analyses

The crude ORs for statin users were 1.14 (95% CI 0.79–1.66) for injuries, 1.10 (95% CI 0.71–1.72) for tendon- or ligament-related injuries, and 1.15 (95% CI 0.69–1.91) for muscle-related injuries compared with controls (Fig. 2). Even after adjustment for age, sex, BMI, and MET hours per week, the ORs for injuries (OR 1.11 [95% CI 0.76–1.62]), tendon- or ligament-related injuries (OR 1.06 [95% CI 0.68–1.66]), and muscle-related injuries (OR 0.98 [95% CI 0.58–1.64]) did not differ between statin users and controls. The risk for exercise-related injuries was also comparable between statin users and non-statin users with hypercholesterolemia. Sub-analysis for the potency of statin use in atorvastatin equivalents did not show a significant dose–response relation for injuries in general (OR 1.06 [95% CI 0.96–1.18] per equivalent), tendon- or ligament-related injuries (OR 1.05 [95% CI 0.93–1.17] per equivalent), or muscle-related injuries (OR 1.02 [95% CI 0.89–1.16] per equivalent). Furthermore, we found no effect of the duration of statin use on the prevalence of injuries.

## Discussion

The present study investigated whether statin use was associated with the prevalence of exercise-related injuries in a large and heterogeneous cohort of amateur runners (*n* = 4460). Injuries were highly prevalent (38%) among participants, but we did not find a higher injury risk in statin-using runners versus control runners (OR 1.14 [95% CI 0.79–1.66]). Sub-analyses revealed a similar lack of relationship between tendon-, ligament-, and muscle-related injuries. There was also no significant association between the potency of statin used or treatment duration and injury risk. These findings demonstrate that the risk for exercise-related injuries is similar between amateur runners regardless of statin use.

A total of 378 amateur runners with a diagnosis of hypercholesterolemia participated in the present study. As expected [17], participants with hypercholesterolemia had a worse cardiovascular risk profile than controls, which justifies the prescription of statins (Table 1). Surprisingly, only 31% (*n* = 117) of the participants with hypercholesterolemia reported using statins. Physically active low-risk individuals may have received lifestyle advice only instead of immediately commencing statin treatment for primary prevention [18]. Alternatively, these runners may not have tolerated statins and discontinued statin therapy. Statin users demonstrated a significantly higher BMI and prevalence of CVD, hypertension, and diabetes mellitus than non-statin users with hypercholesterolemia. These observations demonstrate that statin therapy was appropriately used more often in higher-risk individuals.

Contrary to our hypothesis, statin use did not increase injury risk in amateur runners. The 1-year injury prevalence (38%) with statin use was similar to that observed by others (35%) [19], but crude and adjusted ORs demonstrated no significant difference in injury risk across our three study groups. These findings contrast with data from a large American study (*n* = 46,249) that included active-duty soldiers, veterans, and their family members [5]. Statin users reported an increased risk (OR 1.13 [95% CI 1.05–1.21]) for musculoskeletal injuries during 5 years of follow-up. However, the incidence of musculoskeletal injuries is known to be higher in soldiers than in the general population [20–22] and may also originate from non-exercise activities (e.g., changes in psychological and work demands, increasing rates of depression, and increased symptom awareness and reporting). Our findings do not confirm that statin use increases the prevalence of musculoskeletal injuries; however, the sample size of our study is considerably smaller, which may reduce the statistical power, and exercise amount and intensity was likely greater in the soldiers. A high volume of exercise exposure may be a risk factor for statin-associated symptoms because statin-associated musculoskeletal complaints have previously been reported in highly active individuals [9] and professional athletes [10]. Our cohort was quite active and reported a median exercise volume of 32 MET hours per week, which is equivalent to ~3 times the World Health Organization (WHO) recommendations for 150 min/week of moderate-intensity activities [23] but possibly not active enough to produce a higher rate of complaints.

### Clinical Relevance

We found no evidence for statin-induced exercise-related injuries in a large and heterogeneous cohort of recreational athletes. This observation supports the prescription of statins for primary and secondary prevention of adverse cardiovascular outcomes in physically active individuals with increased cardiovascular risk. In fact, the combination of statin therapy and physical activity yields greater risk reductions for all-cause mortality compared with statin use or physical activity independently [24]. Furthermore, another study found no detrimental effects of statin use on aerobic exercise performance among middle-aged athletes [25]. These findings emphasize that amateur athletes should not fear an increased risk of statin-associated side effects.

### Limitations

There are limitations to the present report. We examined a large population-based cohort of recreational runners, but this cohort included only 117 statin users. We noted that 69% of runners reported having been diagnosed with hypercholesterolemia but were not receiving statins. This raises the possibility that these runners had discontinued statin use because of side effects, but we did not query our cohort about statin discontinuation. Another explanation for the absence of an effect might be explained by runners who stopped running because of statin-induced injuries and therefore did not participate in the Seven Hills Run. This might cause selection bias, which underestimates the true effect. However, this study examined injury prevalence during the year before participation in the study. Our results also depend entirely on self-reported exercise habits and medical diagnoses, which were not confirmed by examining medical records. Further, we assumed that reported injuries were exercise related. Despite these limitations, this study is, to our knowledge, the first to directly examine the relationship between vigorous exercise such as running and possible statin-associated musculoskeletal side effects.

## Conclusion

We did not find an association between statin use in runners and injuries or tendon-, ligament-, and muscle-related injuries. Given the lower risk of cardiovascular events in individuals who are both using statins and have greater cardiorespiratory fitness, runners receiving statins should continue both statin use and their physical activity without undue concern for the risk of injury.
